# Measuring Leukocyte Adhesion to (Primary) Endothelial Cells after Photon and Charged Particle Exposure with a Dedicated Laminar Flow Chamber

**DOI:** 10.3389/fimmu.2017.00627

**Published:** 2017-06-01

**Authors:** Nadine Erbeldinger, Felicitas Rapp, Svetlana Ktitareva, Philipp Wendel, Anna S. Bothe, Till Dettmering, Marco Durante, Thomas Friedrich, Bianca Bertulat, Stephanie Meyer, M. C. Cardoso, Stephanie Hehlgans, Franz Rödel, Claudia Fournier

**Affiliations:** ^1^Department of Biophysics, GSI Helmholtz Center for Heavy Ion Research, Darmstadt, Germany; ^2^Department of Biology, Technical University Darmstadt, Darmstadt, Germany; ^3^Department of Radiotherapy and Oncology, University of Frankfurt, Frankfurt, Germany

**Keywords:** endothelial cells, primary lymphocytes, shear stress, inflammation, adhesion, (particle) irradiation, image analysis, image segmentation

## Abstract

The vascular endothelium interacts with all types of blood cells and is a key modulator of local and systemic inflammatory processes, for example, in the adhesion of blood leukocytes to endothelial cells (EC) and the following extravasation into the injured tissue. The endothelium is constantly exposed to mechanical forces caused by blood flow, and the resulting shear stress is essential for the maintenance of endothelial function. Changes in local hemodynamics are sensed by EC, leading to acute or persistent changes. Therefore, *in vitro* assessment of EC functionality should include shear stress as an essential parameter. Parallel-plate flow chambers with adjustable shear stress can be used to study EC properties. However, commercially available systems are not suitable for radiation experiments, especially with charged particles, which are increasingly used in radiotherapy of tumors. Therefore, research on charged-particle-induced vascular side effects is needed. In addition, α-particle emitters (e.g., radon) are used to treat inflammatory diseases at low doses. In the present study, we established a flow chamber system, applicable for the investigation of radiation induced changes in the adhesion of lymphocytes to EC as readout for the onset of an inflammatory reaction or the modification of a pre-existing inflammatory state. In this system, primary human EC are cultured under physiological laminar shear stress, subjected to a proinflammatory treatment and/or irradiation with X-rays or charged particles, followed by a coincubation with primary human lymphocytes (peripheral blood lymphocytes (PBL)). Analysis is performed by semiautomated quantification of fluorescent staining in microscopic pictures. First results obtained after irradiation with X-rays or helium ions indicate decreased adhesion of PBL to EC under laminar conditions for both radiation qualities, whereas adhesion of PBL under static conditions is not clearly affected by irradiation. Under static conditions, no radiation-induced changes in surface expression of adhesion molecules and activation of nuclear factor kappa B (NF-κB) signaling were observed after single cell-based high-throughput analysis. In subsequent studies, these investigations will be extended to laminar conditions.

## Introduction

The physiological response of the endothelium to inflammatory signals is a graded process of rolling, tight binding, and finally extravasation of leukocytes into inflamed tissue sites that comprise the initial step of the inflammatory cascade (1–3). As opposed to the enhanced adhesion of leukocytes contributing to an inflammatory context, a reduced recruitment of leukocytes to the endothelial layers was found to attenuate inflammatory damage in rodent intestine, brain, and heart (4–6).

Under physiological conditions, the endothelium is exposed to laminar shear stress, which is exerted by the blood flow. These hemodynamic forces determine the functional properties of the endothelium and contribute to the integrity of the blood vessel wall (7). For the assessment of an inflammatory response, the adhesion of leukocytes, i.e., peripheral blood lymphocytes (PBL), to endothelial cells (EC) is used as a read-out *in vitro* and *in vivo* (8, 9). Evidence has been provided that for *in vitro* experimental setups, the integration of physiological, steady laminar flow, as found in microvasculature, or dynamic, non-linear shear stress into culture conditions yields different results compared to static conditions where laminar flow is absent (10, 11). We hypothesize that this accounts even more, when primary cells are used instead of established cell lines in order to be more close to the physiological situation.

In the work presented here, we aim to establish a device which allows for mimicking the blood flow and physiological shear stress to investigate the influence of ionizing radiation on adhesion of PBL to EC *in vitro*. In contrast to therapeutic doses delivered during tumor treatment, exposure to low doses of photons (<6 Gy, in multiple fractions) or low numbers of α-particles (emitted during radon decay, estimated at 2 mSv for one regimen of serial applications) are used successfully for anti-inflammatory treatment of rheumatic and other chronic bone and inflammatory diseases (12–14).

For low-dose radiation therapy, there is growing evidence for an anti-inflammatory effect [reviewed in Ref. (15, 16)]. Modulation of inflammatory cascades has been reported in particular for musculoskeletal diseases (17, 18) and experimentally in *in vitro* and animal studies (9, 14), but the underlying mechanisms are not entirely resolved. One hypothesis to explain the clinical observations is a modulation of the inflammatory response via changes in the interaction of leukocytes with the endothelium by EC on a cellular and molecular level. A well-described key modulator of the inflammatory response is the transcription factor complex nuclear factor kappa B (NF-κB) (19–23). One of its most investigated components is p65/RelA that translocates into the nucleus upon activation by inflammation stimuli such as TNF-α (19, 24, 25). NF-κB signaling is activated in response to irradiation, albeit dependent on the dose range and the cell type (19). However, it has been shown that activation of NF-κB affects the expression of adhesion molecules, thus altering the cell surface to induce PBL binding (26–28).

The cellular and molecular mechanisms elicited by high linear energy transfer (LET) α-particles, however, are barely known. Radon is a radioactive noble gas, evaporating from rocks, and the major dose contribution arises from the emission of α-particles. In contrast to sparsely ionizing X-rays, α-particles are densely ionizing and have a higher relative biological effectiveness for effects that are related to DNA damage, often also with differences in the quality of damage induced (29, 30). However, the tissue response to densely ionizing irradiation, including the interaction of irradiated and non-irradiated cells is less well investigated (31). To unravel the effects of low dose radon exposure, there is a need to investigate the modulation of immune-related responses and inflammation after exposure to densely ionizing irradiation, in particular to α-particles. The reason is that mechanisms considered to be related to X-ray-induced effects are not necessarily transmissible to the α-particle emitter radon due to the abovementioned differences in ionizing density and, as a consequence, to the quality of the DNA damage induced.

For adhesion assays following particle irradiation, performed under physiological laminar culture conditions, commercially available systems (parallel plate flow chambers) are not appropriate for two major reasons. First, when cultivating the cells under laminar conditions prior to irradiation, the scaffold where the cells are attached must be removable to adjust their positioning to the geometry of the beam lines (32), in particular horizontal beams. Second, for low energies, free access of the beam to the cells is necessary to avoid partial or complete shielding from irradiation, because the short penetration depth of the particles is strongly limiting the thickness of the material to be traversed before reaching the cells. In case of low energy α-particles (5.49 MeV), it is around 42 µm in water [calculation according to Ref. (33)].

Here, we report on the establishment of a system with defined laminar flow conditions suitable for the use at particle accelerator facilities (e.g., α-particles or heavy ions). The original flow chamber design (8) included only one chamber and consequently could only monitor one treatment condition at a time. Such systems are currently used for live-imaging during adhesion processes (34), where one flow chamber is set up under a microscope. We constructed a system, where up to five treatment conditions with triplicates (15 dishes with cells in total) can be cultured in parallel in an incubator. In order to be able to analyze a larger number of pictures and to avoid a possible bias of manual counting, we developed a semiautomated, software-based analysis method to evaluate the data from PBL adhesion assays.

## Materials and Methods

### Cultivation of Human Microvascular Endothelial Cells (HMVEC)

Human microvascular endothelial cells were purchased from Cell Applications Inc. (San Diego, CA, USA). The cells were maintained in VascuLife EnGS-Mv Medium Complete Kit (PELOBiotech GmbH) according to the supplier’s instructions. Change of medium was performed thrice a week, and cells were passaged when reaching ~85% of confluence. For experiments, cells were used at passage numbers between 5 and 8. The expression of typical endothelial marker CD34 was tested by immunocytochemistry, and absence of the smooth muscle marker α-SMA was confirmed to rule out the contamination of cultures with smooth muscle cells from initial preparation (data not shown).

For nuclear p65 translocation experiments, HMVEC were seeded into 96-well plates (ViewPlate-96 Black, PerkinElmer, Waltham, MA, USA) at 1 × 10^4^ cells/mL (2,000 cells/well) and cultivated 48 h before start of the experiments (stimulation alone or in combination with X-irradiation). In parallel, HMVEC cultures were kept without treatment to precondition medium, which was used to change the medium after treatment of the samples.

### Cultivation of Ea.hy926 (Human Hybrid Endothelial Cells)

Ea.hy926 were purchased from ATCC (ATCC^®^ CRL-2922™; LGC Standards GmbH, Wesel, Germany) and maintained in Dulbecco’s modified Eagles medium supplemented with 10% FBS and 1% penicillin–streptomycin (all from Biochrom, Berlin, Germany). Change of medium was performed thrice a week, and cells were passaged when reaching ~85% of confluence.

### Setup of Static and Laminar Shear (“Flow”) Cultivation

**Figure 1:**
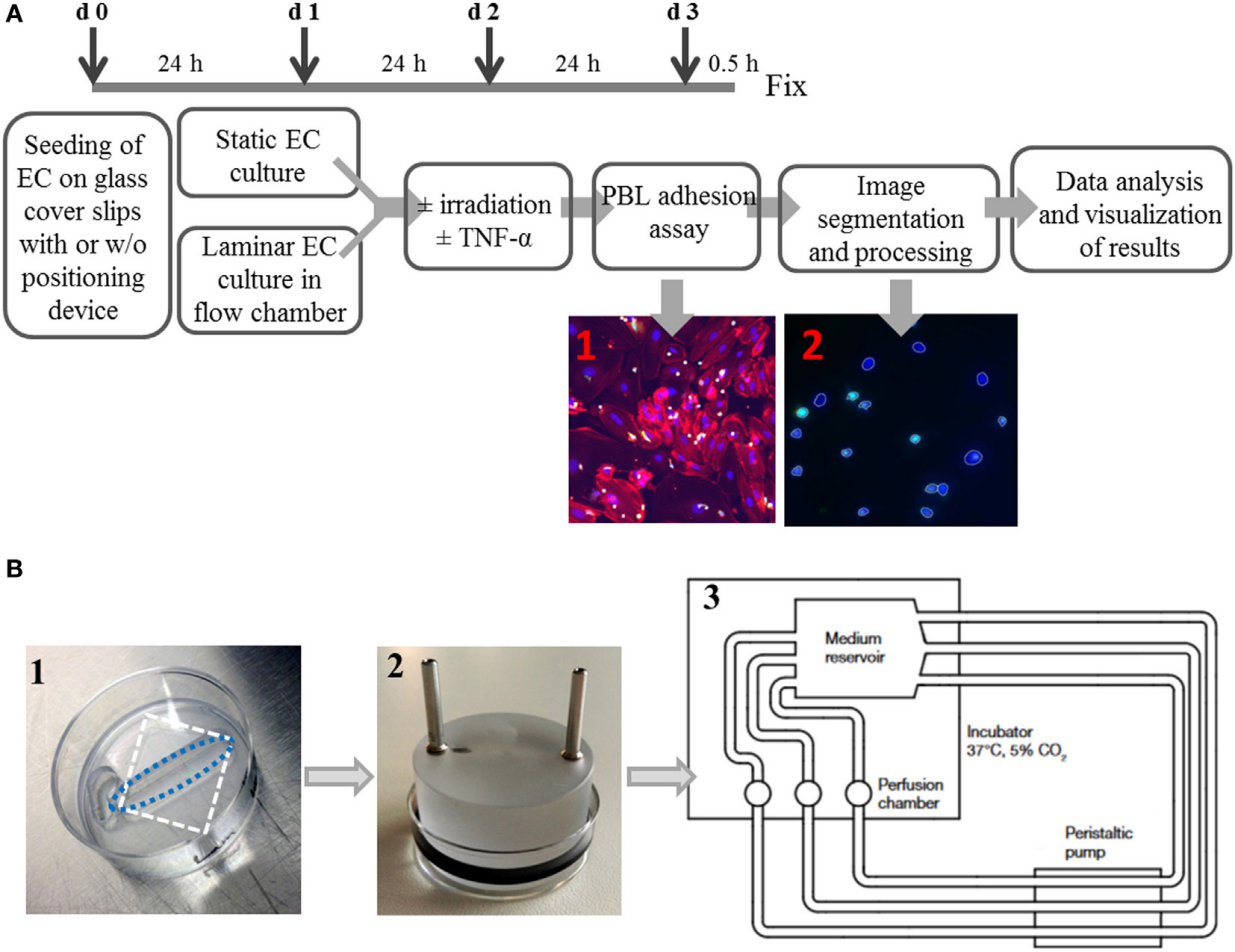
**Experimental setup of a system to culture endothelial cells under static or laminar flow culture conditions and subsequent semiautomated analysis**. Primary human microvascular endothelial cells (HMVEC) were cultured either under *static* conditions or physiological *laminar* flow in a custom-made flow chamber system. The experimental workflow is shown in **(A)**. Cells are seeded with or w/o positioning devices (d0) and pre-cultured for 24 h. Next, the dishes with cells are inserted into the flow chamber (d1) for cultivation under *laminar* conditions for 24 h, while in *static* cultures, only the medium is changed. On day 2, cells were treated with or w/o TNF-α, with or w/o irradiation. After an additional cultivation for 24 h under laminar or static conditions, PBL are coincubated for 0.5 h, fixed and processed for microscopy. The image files are then processed using an ImageJ macro and segmented with freely available software (CellProfiler) to generate mean values of the replicates. The detailed setup of the flow chamber is shown in **(B)**: (1) seeding of cells with positioning device, white dashed rectangle outlines the cover slip; blue dashed oval outlines the growth area of the cells, (2) application of perfusion insert after attachment of cells, and (3) flow chamber scheme.

An overview over the experimental setup is given in Figure 1A; the details of the flow chamber are shown in Figure 1B.

Human microvascular endothelial cells were seeded onto fibronectin coated (Merck, Darmstadt, Germany; 0.1 µg/mL in PBS; 0.06 ng/mm^2^) autoclaved glass cover slips (Carl Roth, Karlsruhe, Germany) placed in 35 mm cell culture dishes (Thermo Scientific Nunclon, Waltham, MA, USA) and cultivated under static or laminar conditions.

#### Static Setting

Human microvascular endothelial cells were seeded at a density of 615 cells/mm^2^. After 2 h, cells had attached to the glass and 1.5 mL of medium was added. Medium was changed the next day to remove cell debris. Two days after seeding, cells were irradiated at various doses. Sham controls underwent the same transport procedure without irradiation. Immediately after irradiation, medium was removed and TNF-α-containing culture medium (R&D Systems, Wiesbaden, Germany; 1 ng/mL, 2 mL per dish) was added, except for negative controls which received normal culture medium. Cells were further cultivated 24 h before proceeding with the adhesion assay.

#### Laminar Setting

**Figure 2:**
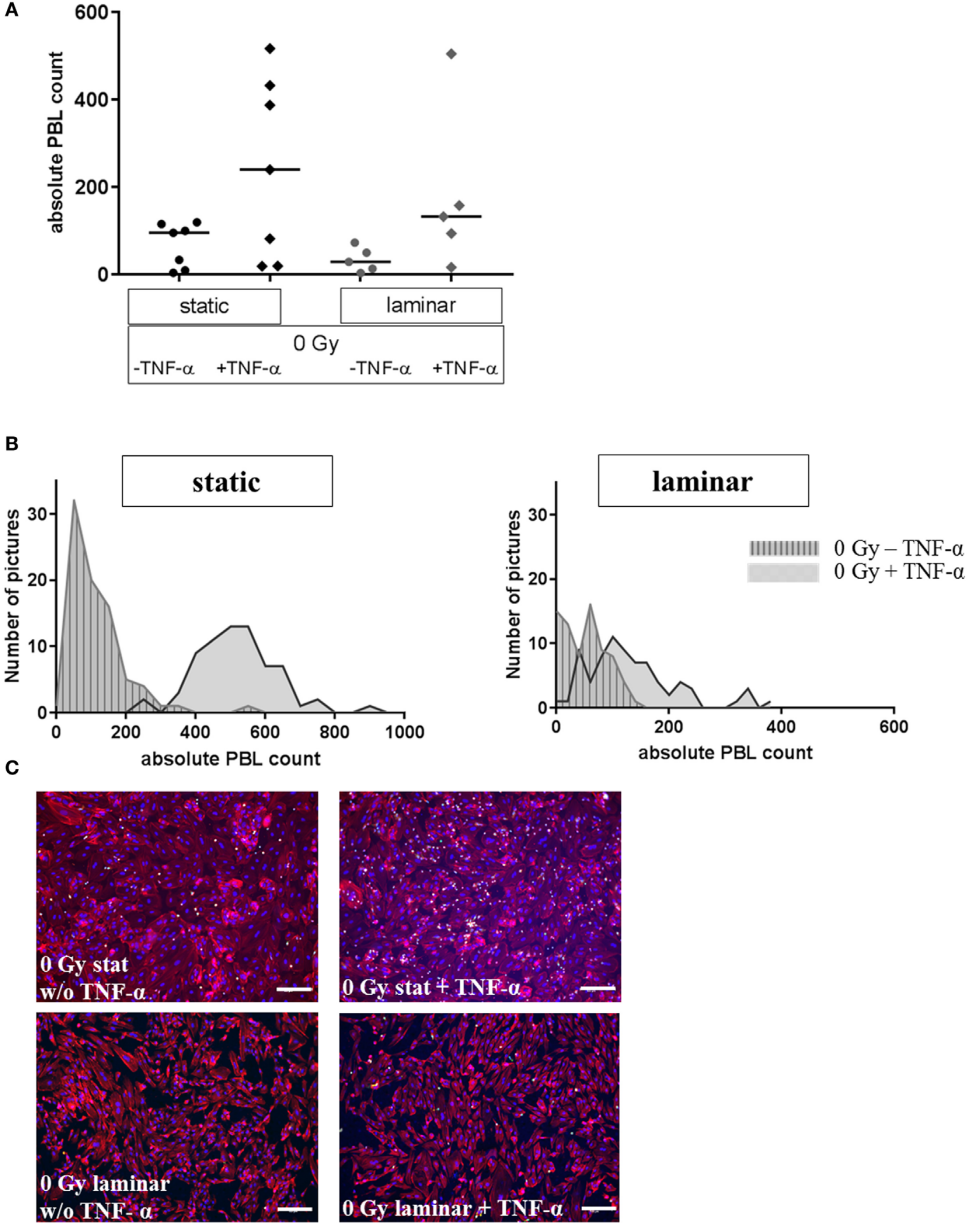
**Adhesion assay of PBL to stimulated human microvascular endothelial cell (HMVEC) under static or laminar flow culture conditions**. Adhesion of PBL to HMVEC is shown under *static* or *laminar* culture conditions, treated with or w/o TNF-α. Absolute counts of adherent PBL to HMVEC are shown in **(A)**; N static = 7, N laminar = 5; duplicates or triplicates were used for each experiment; line = median. Distributions of adherent PBL counts are shown in **(B)** for one representative experiment each; left: static culture conditions, right: laminar culture conditions; gray/striped: untreated, gray: with TNF-α. Representative microphotographs are given in **(C)**. Upper panel: static culture conditions, without (left) or with TNF-α (right); lower panel: laminar culture conditions, without (left) or with TNF-α (right). Blue = DAPI/nuclei, red = TRITC-Phalloidin/cytoskeleton, green = CFDA/PBL; 100×, bars = 100 µm.

**Figure 3:**
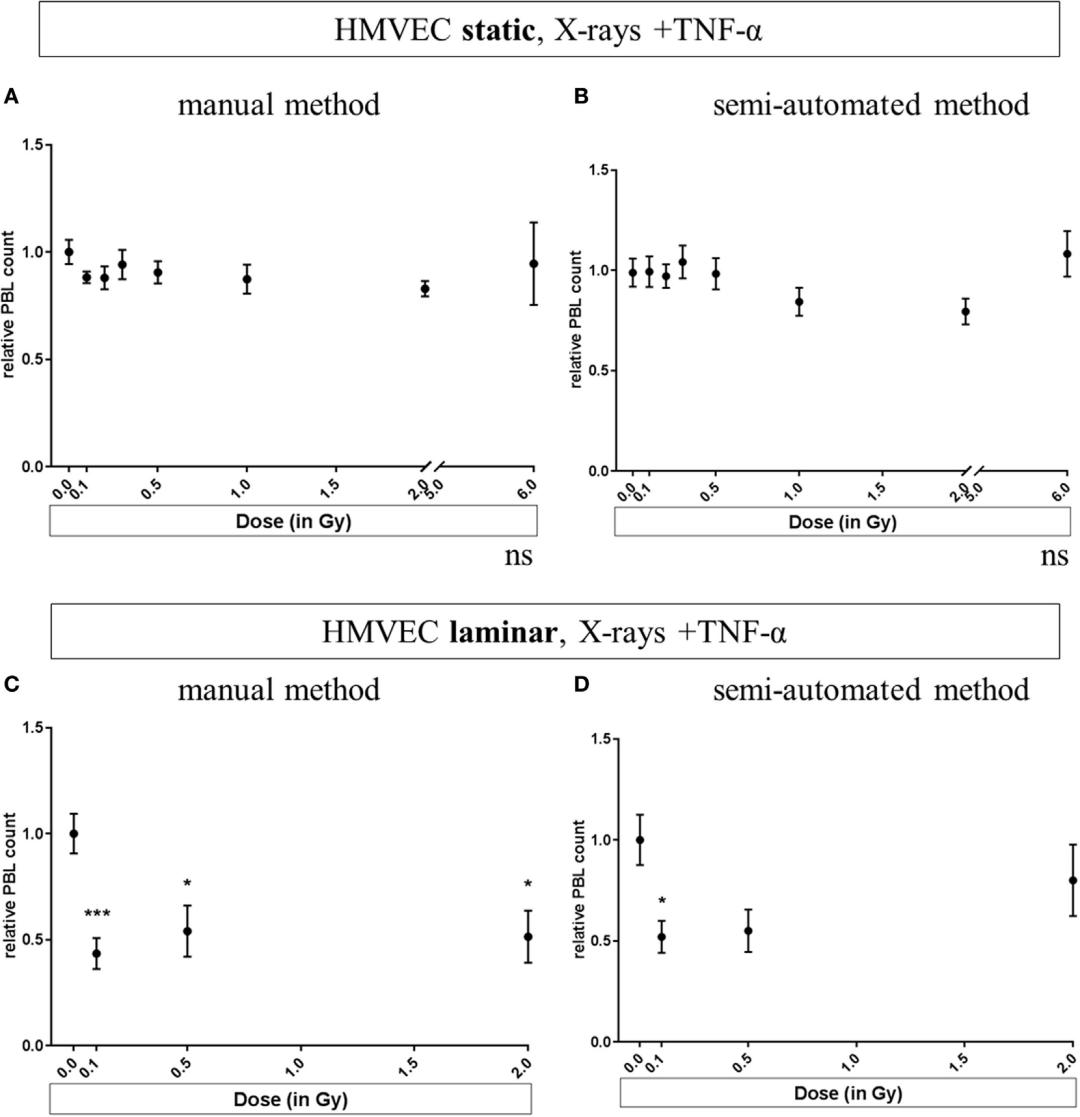
**Effects of low-dose X-irradiation on adhesion of PBL to human microvascular endothelial cell (HMVEC)**. HMVEC were cultivated under static **(A,B)** or laminar **(C,D)** conditions for 24 h, irradiated with X-ray doses ranging from 0.1 to 0.5 Gy, and 2 Gy **(C,D)** or 6 Gy **(A,B)**; stimulated with TNF-α and cocultivated with PBL 24 h afterward. The numbers of attached PBL were normalized to the averaged values obtained after treatment of HMVEC with TNF-α/0 Gy (reference value). The data analysis has been refined during the development of the assay. In initial experiments, PBL were manually counted per visual field using a microscope [**(A)** *static* and **(C)** *laminar*]. Then, a semiautomated method was developed to quantify for each field of view the numbers of both PBL and HMVEC [**(B)** *static* and **(D)** *laminar*]. *N* = 3 for all experiments except for **(A,B)**: 6 Gy (*N* = 1); 0.2 and 0.3 Gy (*N* = 2); for each condition duplicates or triplicates were measured. Replicates of all experiments were pooled and mean values ± SEM were calculated. One-way ANOVA was applied; *p* < 0.05 was considered as significantly different from the reference value and labeled. *p*-values versus 0 Gy + TNF-α in detail: Panel **(C)**: *p* = 0.0009 for 0.1 Gy + TNF-α; *p* = 0.0234 for 0.5 Gy + TNF-α and *p* = 0.0102 for 2 Gy + TNF-α. Panel **(D)**: *p* = 0.0254 for 0.1 Gy + TNF-α; *p* = 0.0942 for 0.5 Gy + TNF-α and *p* = 0.6634 for 2 Gy + TNF-α.

Autoclaved glass cover slips were placed in 35 mm Petri dishes and tightly covered by disposable positioning devices (Warner Instruments, Holliston, MA, USA; slotted, 6 mm × 24.5 mm bath) (Figure 1B, 1). The open area was then coated with fibronectin (0.1 µg/mL in PBS; 0.06 ng/mm^2^) for 1 h in an incubator. After removing the coating solution, cells were seeded at 0.1 × 10^6^ cells/0.5 mL in the open area of the positioning devices. Dishes were incubated for 2 h to allow for attachment of the cells and then 1.5 mL of medium were added for further cultivation. The next day, laminar flow was introduced by connecting each dish with an insert containing two channels for medium in- and outflow (custom-made) and an O-ring (Perbunan, Wollschlaeger GmbH, Bochum, Germany) inserted into a groove at the side of the insert to seal the dish (Figure 1B, 2). The channels were connected with silicone tubes (inner diameter 1.6 mm, Ismatec, Wertheim, Germany), medium reservoirs and a peristaltic pump (Ismatec) for constant medium transport. Each treatment group (triplicates) had its own medium reservoir, consisting of a modified T75-cell culture flask containing 150 mL of culture medium (Figure 1B, 3). Three holes were drilled into the plastic on top and three at one side of the flask, through which stainless steel channels were inserted to attach the tubes to and from the pump. The flow (*ν*) was set to 0.029 m/s, and shear stress (*τ*) was calculated according to the formula:

$$\tau =\frac{6\mu \text{Q}}{bh^{2}}$$

with *Q* = flow rate (*ν* × *b* × *h*), *μ* = fluid viscosity, *b* = width of the chamber, and *h* = height of the chamber [modified after (35)]. Using this approach, we applied a shear stress of 0.75 dyn/cm^2^, which is in the range of physiological blood flow in small vessels (34).

Cells were adapted to laminar flow for 24 h before irradiation. For irradiation purpose, medium flow was stopped, tubes were detached from the dishes, perfusion inserts were removed, and 2 mL of medium was added on top of the positioning device. Then, cells were irradiated and immediately reintroduced into the flow chamber system. Medium was replaced in the flasks by TNF-α-containing culture medium (1 ng/mL), except for negative controls. The cells were further cultivated for 24 h before proceeding with the adhesion assay.

### X-Irradiation

X-irradiation was performed using an X-ray tube (General Electrics, München, Germany) with a cathode current of 16 mA and an acceleration voltage of 250 kV. Cells were removed from the flow chamber, or the incubator for static conditions, respectively, carried to the X-ray tube and irradiated at a dose rate of 1.5 Gy/min. Control samples were subjected to the same mechanical stress and temperature changes. After irradiation, cells were reintroduced immediately to laminar flow, or put back into the incubator, and cultivated further. All samples received a change of medium including 1 ng/mL TNF-α (except for negative controls).

### Helium (He)-Ion Irradiation

Helium-ion irradiation was carried out at the UNILAC facility of GSI Helmholtz Centre for Heavy Ion Research, Darmstadt, Germany. He-ions were used at an energy of 1.62 MeV/u (on target) and a LET of 76 keV/u. Details of the irradiation facility are described elsewhere (32). Immediately before irradiation, laminar cultures were disassembled from the flow chamber, and glass cover slips from static cultures were fixed with autoclaved O-rings. Laminar cultures are fixed by the chamber inserts. All dishes with cells were placed vertically in magazines, which were filled up with basal culture medium. Control samples were treated accordingly and carried along with irradiated samples. After irradiation, cells were reintroduced immediately to laminar flow, or had O-rings removed and were put back into the incubator for further cultivation. All samples received a change of medium including 1 ng/mL TNF-α (except for negative controls).

High LET and the Poisson distribution of the ion traversals per cell imply that for low doses of He-ions not all cell nuclei are hit by a charged particle (Table 1), i.e., at 0.1 Gy 20% of the cell nuclei are not hit. The respective calculations have been performed as follows.

**Table 1:** **Calculation of mean number of hits per nucleus and cells with 0, 1, or 2 hits according to the Poisson distribution, based on the nuclear area (HMVEC) = 200 µm^2^ and LET (He) = 76 keV/µm**.

Dose (Gy)	Fluence (p/cm^2^)	Mean number of hits/nucleus	Cells with 0 hit
0.1	8.21 × 10^5^	1.6	0.2
0.5	4.11 × 10^6^	8.2	2.7 × 10^−4^
1	8.21 × 10^6^	16.5	7.1 × 10^−8^
2	1.64 × 10^7^	32.9	5.1 × 10^−15^

Dose and fluence (particles/cm^2^) are related according to: d

(1) $$\text{Fluence }({\text{p/cm}}^{\text{2}})=\frac{\text{Dose }(\text{Gy})\times 10^{9}}{1.602\times \text{LET }\left(\frac{\text{keV}}{\mathrm{\mu m}}\right)}$$

The mean number of particle hits per nucleus has been calculated based on fluence and mean nuclear area, determined to be about 200 µm^2^ (quantitative analysis of 250 cell nuclei after nuclear staining with DAPI):

(2) Mean number of hits N=Fluence×Nuclear area.

The fraction of cells which were not hit by a charged particle has been calculated based on the Poisson distribution of the number of particle traversals per cell nucleus, according to

(3) $$P_{0}=e^{-N}.$$

### Adhesion Assay with Human PBL

The basic adhesion assay protocol used here was modified according to Kern et al. (36) and adapted to the static or laminar setting. An overview is shown in Figure 1A.

One day before adhesion assay (at the day of irradiation), peripheral blood lymphocytes (PBL) were isolated from human blood obtained from a blood bank (German Red Cross Blood Donor Service, Frankfurt/Main). Whole blood (buffy coat) was diluted 1:1 with PBS^−/−^ and separated by Biocoll (1.077 g/mL; both Merck Millipore, Darmstadt, Germany), based on a protocol published elsewhere (37). The interphase containing lymphocytes was incubated with red blood cell lysis buffer (8.29 g/L NH_4_Cl, 1 g/L KHCO_3_, 37.2 mg/L Na_2_EDTA in aqua dest, pH 7.2) for 5 min to remove erythrocytes and washed. Donor-specific autologous serum was collected, heat-inactivated at 56°C for 30 min and added to the lymphocyte medium (X-vivo 15, Lonza, Basel, Switzerland; 1% penicillin–streptomycin (Biochrom, Berlin, Germany), 3% autologous, heat-inactivated serum). PBL were incubated for 4 h to allow for separation of monocytes via attachment. Then, the supernatant containing non-attached PBL was removed and further cultivated in RPMI 1640 + l-glutamine, 1% HEPES, 1% penicillin–streptomycin and 20% heat-inactivated FBS (all from Biochrom) (“PBL medium”) over night.

The next day, PBL were collected by centrifugation (10 min, 1,000 rpm) and mixed with serum-free RPMI 1640 + glutamine/1% HEPES/1% penicillin–streptomycin (“staining medium,” 50 mL per donor) and 50 µg Cell tracker green (CFDA, 5-chloromethylfluorescein diacetate, Thermo Fisher, Waltham, MA, USA; dissolved in 1 mL DMSO). Cells were allowed to incorporate the dye for 1 h in the incubator, and cell numbers were determined by using a Coulter Counter (Z-series, Beckmann Coulter, Krefeld, Germany). Then, PBL were collected by centrifugation (10 min, 1,000 rpm), mixed with PBL medium at a concentration of 1 × 10^6^/mL and added to the HMVEC. For static assays, 1 mL (1 × 10^6^ cells) was used, for the flow chamber, 30 × 10^6^ cells in 150 mL were used to fill each medium reservoir, connected to the triplicates of one condition. Cell adhesion was allowed for 30 min in the incubator for both static and laminar setting. Then, cells were washed with PBS^−/−^, fixed with 4% formaldehyde (Carl Roth)/PBS^−/−^ for 15 min and stored in PBS at 4°C until staining.

### Staining and Image Acquisition

For visualization, cells were pre-treated with PBS/Triton X100 (Carl Roth, 0.3%) for 10 min and stained with TRITC-Phalloidin (4 µg/mL) and DAPI (4 µg/mL, both from Sigma-Aldrich, Taufkirchen, Germany) in PBS/Triton X100 (0.3%) for 45 min, washed three times with PBS and once with distilled water and mounted on slides with fluorescence-protecting mounting medium (VWR, Auckland, New Zealand).

Adherent PBL were evaluated by a manual or a semiautomated analysis. Using the manual method, PBL were counted in 6 microscopic fields (100× magnification) per replicate, or using the semiautomated analysis, PBL number was determined in 15–25 microphotographs per replicate, taken on an epifluorescence microscope (Leica DMI 4000B) equipped with a monochrome camera (DFC360 FX) with a 100× magnification (ACS APO 10x/0.30 CS, all from Leica, Wetzlar, Germany). The pictures were stored and analyzed as described subsequently.

### Image Analysis

For semiautomated image analysis, microscope files acquired with a Leica DMI 4000 (“.lif”-format) were split into single channels using a customized ImageJ macro. ImageJ is freely available [https://imagej.net/Downloads (38)]. The macro is available upon request. Single channels were saved as gray-value.tiff files with 12bit depth. The .tiff-files were then loaded into CellProfiler software, which is also freely available [http://cellprofiler.org (39)], and analyzed for total cell number. To this end, DAPI-positive nuclei from pictures showing the blue channel were segmented, and PBLs were segmented by CFDA fluorescence from pictures showing the green channel. Due to their different shape, a diameter of 3–35 pixels for PBL, and 3–80 pixels for nuclei was chosen. The number of PBL was determined and used for further analysis. In some rare cases, the software was not able to segment cells correctly, e.g., due to staining artifacts. Those images were excluded from analysis. In parallel, the number of EC was determined to control for possible large deviations, and samples were checked for uniform growth of EC as a quality control. Within the range of EC numbers/picture measured in the experiments, no significant correlation with adhesion, i.e., the detected number of PBL/picture was found; therefore, this parameter was not included in the analysis.

### Statistical Analysis of the Adhesion Data

The number of PBL/picture was measured, typically in triplicates of three to four independent experiments (number of experiments = *N* is depicted in the figure legends). For statistical analysis, the mean number of PBL/picture was determined for each replicate separately. The reference value for each experiment (0 Gy/ + TNF-α) was calculated by averaging the values of the respective replicates. The values for the irradiated replicates were normalized to the reference values of the respective experiments. For each data point, mean values and SEM over all replicates of all experiments were calculated. Statistical significance was tested using ANOVA or Student’s *t*-test. Graphs were generated using Prism Ver. 6 and 7 (GraphPad, LaJolla, CA, USA).

### Flow Cytometric Measurement of Adhesion Molecules

Endothelial cells were irradiated and TNF-α stimulated as described for the adhesion assay under static conditions. After treatment, cells were detached (citric saline, 1.35 M KCl, 0.15 M sodium citrate) after 24 h and fluorescence intensities of fluorochrome-conjugated antibodies directed against ICAM-1-PE (BD Biosciences, Heidelberg, Germany), VCAM-1-APC, E-Selectin-FITC, or of the isotype control (IgG1-PE) (all R&D Systems, Wiesbaden, Germany) were measured by flow cytometry (Partec PAS III, Partec, Muenster, Germany). Cells were gated for their population characteristics in the FSC-SSC-plot, and the mean fluorescence intensity of the respective fluorochromes was calculated using appropriate software packages (FloMax1^®^-software, Partec). In each experiment and for each condition, at least triplicates were measured.

### Cultivation and Treatment of HMVEC for NF-κB (p65 Subunit) Nuclear Translocation Measurements

Following irradiation as described above, medium was changed using the conditioned medium, obtained in parallel cultures, with or w/o TNF-α (1 ng/mL). After 0, 1, 3, and 24 h, cells were fixed in 4% (v/v) formaldehyde solution/PBS (10 min) and processed for immunohistological staining (washing 3×, permeabilization in PBS/0.5% (v/v) Triton X-100 for 15 min, washing 3×, blocking in PBS/4% (w/v) BSA for 30 min). For the detection of NF-κB signals, the following antibodies were used: primary monoclonal rabbit anti-NF-κB antibody [anti-NF-κB-p65 RabMab, #ab76311, Epitomics, Burlingame, CA, USA; 1:500 in 1% (w/v) BSA/PBS, incubation for 3 h] and donkey anti-rabbit IgG Cy3-conjugated secondary antibody [#711-165-152, Jackson, West Grove, PA, USA; 1:500 in 1% (w/v) BSA in PBS, incubation for 1 h]. Nuclei were counterstained with DAPI, and Alexa-488-conjugated phalloidin (1:200, Invitrogen, Carlsbad, CA, USA) was used as a cytoskeleton marker (actin), respectively. Finally, samples were briefly washed in H_2_O and subsequently mounted in Mowiol 4-88 mounting medium (Sigma-Aldrich).

### High Content (HC) Imaging, Image Analysis, and Statistical Analysis

High content image analysis was performed using the “Operetta” HC imaging system (PerkinElmer Cellular Technologies Germany GmbH, Hamburg, Germany) with built-in “Harmony” analysis software and “phonologic” extension package. Per cavity (96 flat bottom plate) and condition, 5 × 5 images were acquired using a 20× long working distance objective (NA 0.45; focus depth 4.6 µm; pixel size: 0.496 µm/pixel) and suitable filter settings for Cy3, Alexa 488, and DAPI signals. After image acquisition, the built-in Harmony software was used to generate an analysis routine that allows discriminating subpopulations based on nuclear morphology parameters (Figure S3 in Supplementary Material). Therefore, the software was trained by user-assistance to distinguish between artifacts and three major nuclear classes with the following characteristics: (A) medium-sized nuclei, rather oval than round, (B) large nuclei, rather round or oval, (C) small nuclei, rather elongated or oval (Figure S3 in Supplementary Material). For each cell, median p65 fluorescence intensities inside the nucleus [median I (A_nuc_)] and within a defined cytoplasm area around the nucleus [median I (A_cyto_)] were calculated and utilized to obtain median ratios of relative nuclear p65 fold-changes. Without considering ~15% of nuclei (“trash,” see (Figure 6A), the average number of evaluated nuclei per replicate and condition ranged for class A between 480 and 1,800 and for classes B and C between 400 and 1,000 nuclei, respectively. The resulting numeric values were fed into the open source software R (http://www.R-project.org/) for further statistical analysis and visualization. Tukey box plots were generated for each morphological class and condition, showing the relative nuclear fold-change normalized to unirradiated controls. All experiments were performed in duplicates.

## Results

### Application of Laminar Flow to Human Primary Endothelial Cells (HMVEC) in Irradiation Experiments

It is considered important to perform studies on EC under physiological laminar flow conditions (40, 41). These experiments, especially at ion accelerators, require an open access to the cell layer and to be adjustable to beam exit window geometry, e.g., vertical or horizontal positioning of samples, maximum sample dimensions for homogenous dose distribution, or penetration depth of ion beams (32). For this reason, we improved the basic flow chamber model by Freyberg and Friedl (8) to meet these requirements. For this study, we used human primary endothelial cells (HMVEC). In principle, other EC types or any adherent cell type can also be used in the flow chamber system.

### Workflow of Adhesion Assay and Data Analysis

We framed an experimental setup, shown as an overview in Figure 1A. Seeding, cultivation under different conditions, irradiation and adhesion assay are described in Materials and Methods section. In order to analyze PBL adhesion to HMVEC, we developed a method for semiautomated image analysis. Before this analysis protocol had been introduced, the scoring evaluator identified and counted adherent PBL by their green CFDA-fluorescence per visual field. The number of EC was not taken into consideration. In the advanced protocol, software-based segmentation and image analysis was developed. This improved method of analysis also allowed for a higher throughput of pictures and reduced possible bias of the evaluator. With the first method, 6 pictures were evaluated per replicate, and only the number of PBL was recorded. With the semiautomated method, all pictures were recorded, so that a re-evaluation at later time points is possible.

In our system, up to 15 Petri dishes with EC (HMVEC), representing different conditions or replicates, can be cultivated at a time. Each replicate was kept in a separate cell culture dish (Figures 1B, 1,2), and up to three dishes were connected to one shared medium reservoir (Figure 1B, 3). For irradiation setup, dishes can rapidly be removed from the system, taken to the experimental lab, and assembled back into the flow chamber to continue cultivation. The readout used here to study HMVEC activation (by TNF-α stimulation) was lymphocyte (PBL) adhesion after combined treatment with TNF-α and irradiation. Accordingly, TNF-α-stimulated, non-irradiated EC served as a positive control. Irradiated and TNF-α-treated samples were normalized to the respective control value (0 Gy + TNF-α). The number of PBL per picture was determined by image analysis of the single channels and segmentation of the images.

### Comparison of TNF-α Stimulation of HMVEC under Static or Laminar Flow Culture Conditions

First, the influence of stimulation by the proinflammatory cytokine TNF-α on the adhesion of PBL to HMVEC was investigated comparing static and laminar conditions. As shown in Figure 2, under static culture conditions binding of PBL to mock-stimulated HMVEC (without TNF-α) was more than two times higher as compared to HMVEC cultured under laminar flow conditions without TNF-α (median for static culture conditions: 95, median for laminar conditions: 29, Figure 2A). When stimulated with TNF-α, PBL binding increased about 2.5-fold under static conditions (median: 240), and about 5-fold for laminar conditions (median: 132). The distribution of PBL for static conditions was broader compared to laminar conditions, where more data points accumulate close to the median.

The distribution of the values of representative data points are depicted in Figure 2B, showing that for TNF-α-treated cells lower values occur under laminar flow compared to the corresponding samples cultured under static conditions. In addition, the distribution indicates a considerable heterogeneity within one data point. Representative photomicrographs (Figure 2C) showed that the morphology of HMVEC was more cobblestone-like under static conditions (upper panel), whereas the cells were more elongated under laminar flow (lower panel).

### Effect of (Low-Dose) X-Irradiation to HMVEC under Static or Laminar Flow Conditions

Results obtained for static or laminar culture conditions in combination with TNF-α stimulation and X-irradiation of HMVEC are depicted in Figure 3. In addition to the use of two culture methods, two different methods of analyzing PBL adhesion were investigated: the manual counting method (Figures 3A,C) and the refined, semiautomated method (Figures 3B,D).

In Figure 3A, pooled experiments for static conditions, analyzed with the manual method are depicted, indicating no significant changes after TNF-α stimulation and X-irradiation. The same was observed when using the semiautomated method (Figure 3B).

For Figures 3A–D, the same experiments were analyzed respectively. As a validation of the semiautomated method, randomly chosen pictures of the experiments presented in (Figures 3B,D) were verified visually (data not shown).

In Figure 3A, pooled experiments for static conditions analyzed with the manual method are shown. No significant changes were found after TNF-α stimulation and X-irradiation. The same was observed when using the semiautomated method (Figure 3B). In contrast, for laminar culture conditions, the radiation induced reduction of PBL adhesion was confirmed for low and intermediate doses (Figure 3C for the manual counting method, Figure 3D for the semiautomated analysis). With both methods, a clear decrease of PBL adhesion after exposure to low doses (0.1 and 0.5 Gy + TNF-α) to 0.4–0.5 was detected, while the effect for the higher dose (2 Gy + TNF-α) was not clear. Under laminar conditions, five treatments including controls can be applied at a time. To focus on the effects in response to lower doses as used in anti-inflammatory therapy, we chose to test doses from 0.1 to 2 Gy under laminar conditions in subsequent experiments (Figures 3C,D).

### Effect of (Low-Dose) He-Ion Irradiation on HMVEC with and without Laminar Flow Conditions

**Figure 4:**
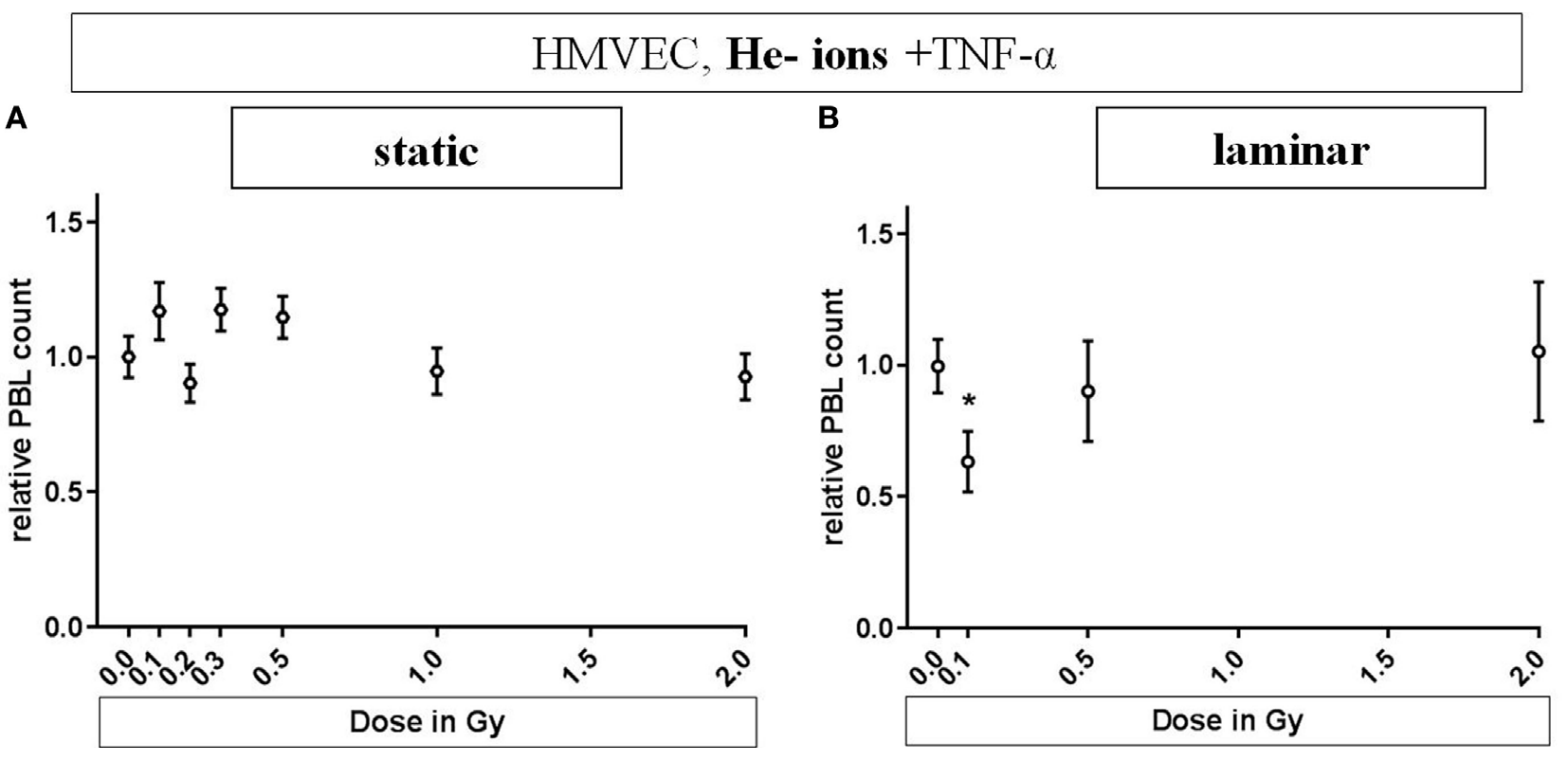
**Effects of low-dose He-ion irradiation on PBL adhesion to human microvascular endothelial cell (HMVEC)**. HMVEC were cultivated under static **(A)** or laminar **(B)** conditions for 24 h, irradiated with different doses of He-ions [ranging from 0.1 to 2 Gy, linear energy transfer (LET) 76 keV/u], stimulated with TNF-α and cocultivated with PBL 24 h later. The numbers of attached PBL were normalized to the values obtained after treatment of EC with TNF-α/0 Gy (reference value). Experiments were analyzed with the semiautomated method described above. **(A,B)**: *N* = 3. Mean ± SEM. *t*-Test was applied; only the value for TNF-α/0 Gy compared to TNF-α/0.1 Gy under laminar conditions was significant (*p* = 0.027).

Human microvascular endothelial cells were cultivated under static or laminar conditions, stimulated and irradiated with different doses of He-ions (0.1 to 2 Gy, Figure 4). We did not observe cell loss due to transportation to the heavy ion irradiation facility (data not shown). Under static conditions (Figure 4A) PBL adhesion fluctuates around the reference level. Under laminar conditions (Figure 4B), adhesion was lowered to about 0.6 at a low dose of 0.1 Gy (*p* = 0.027) and was comparable to reference levels after exposure to 0.5 or 2 Gy of He-ions + TNF-α.

### Expression of Adhesion Molecules on the Surface of HMVEC after Stimulation with TNF-α and Exposure to Low Doses of X-Irradiation

**Figure 5:**
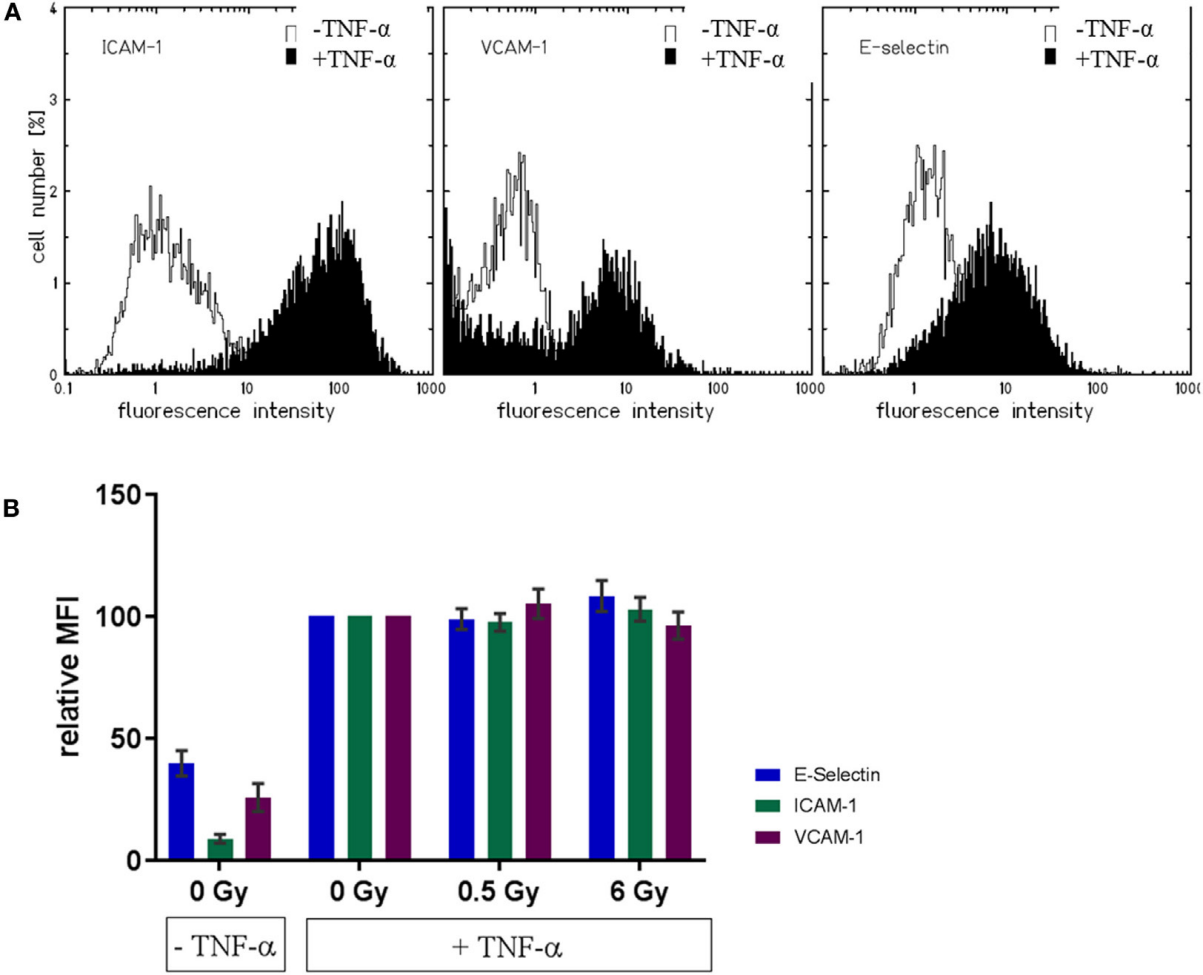
**Expression of adhesion molecule on the cellular surface of primary human microvascular endothelial cell (HMVEC) (static conditions)**. Representative distributions of fluorescence intensities for adhesion molecules in unirradiated HMVEC **(A)** are shown, either without (white curves) or with TNF-α (black curves). Mean fluorescence intensities for the respective molecules after irradiation are depicted in **(B)**, with baseline levels (TNF-α/0 Gy) and levels of treated cells (with TNF-α, with or w/o irradiation) (*N* = 3).

Next, we aimed to investigate factors reported to be associated with changes in adhesion observed after TNF-α stimulation and irradiation (36). Under static conditions, the measurement of the expression of adhesion molecules on the cellular surface of mock-irradiated HMVEC revealed, that the basal expression levels of adhesion molecules, ICAM-1, VCAM-1, and E-Selectin, were enhanced after TNF-α treatment. This can be inferred from representative distributions shown in Figure 5A and is reflected by the mean fluorescence intensities (Figure 5B). However, radiation induced modifications have not been detected compared to TNF-α treatment only (Figure 5B). Also, almost complete overlap with the TNF-α-treated control samples was detected in the distribution of the fluorescence intensities (not shown).

### NF-κB Nuclear Translocation after TNF-α Stimulation and/or X-Irradiation of Human Primary EC (HMVEC) under Static Conditions

**Figure 6:**
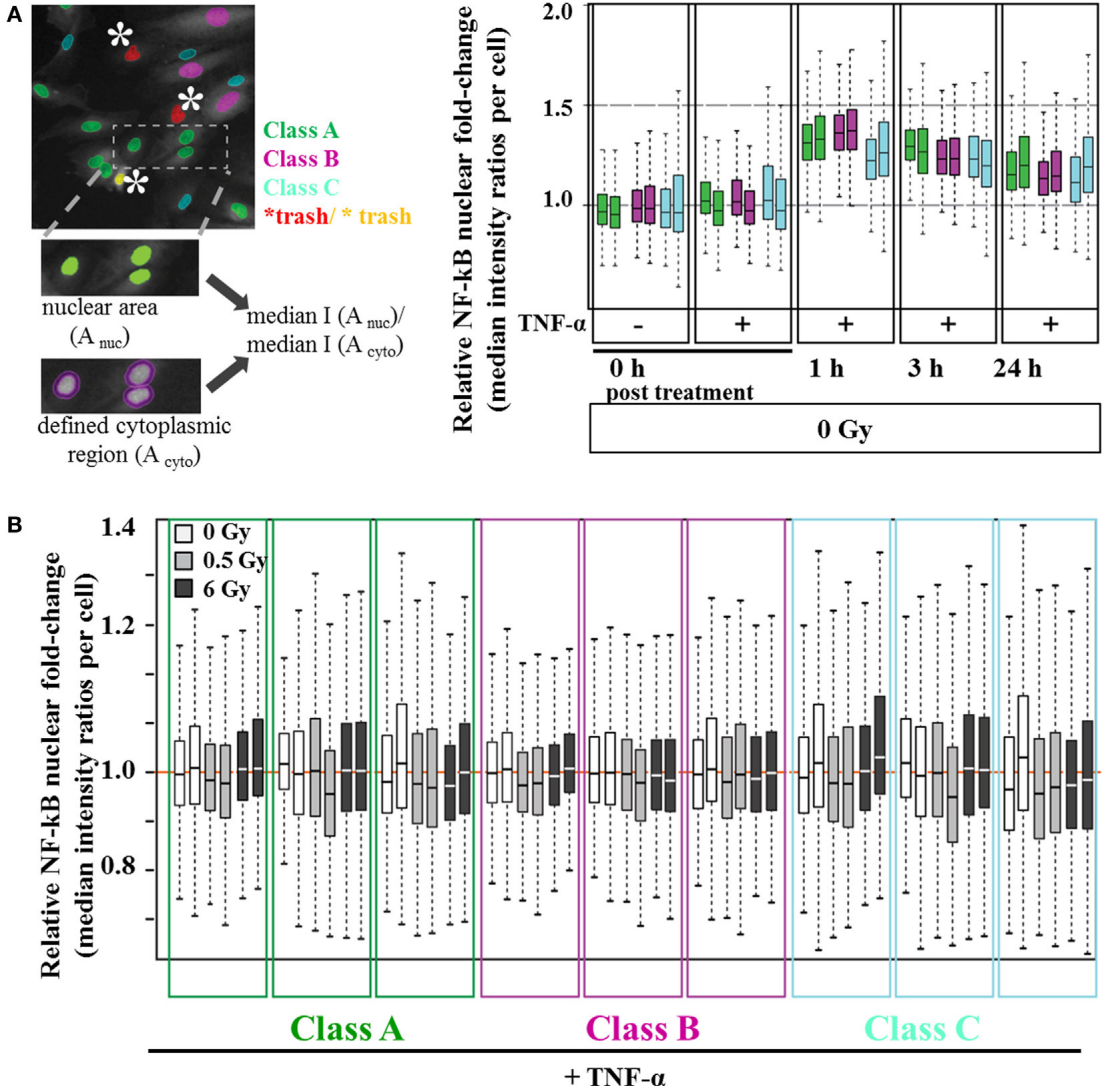
**Nuclear NF-κB translocation after combined treatment with TNF-α and irradiation**. Human microvascular endothelial cells (HMVEC) were classified into subpopulations based on nuclear morphology [**(A)**, left panel, see also Figure S3 in Supplementary Material]. Relative nuclear p65 fluorescence intensity was calculated for each class per nucleus as the ratio of single nuclei median intensity I (A_nuc_) over single cytoplasm median intensity I (A_cyto_). To obtain relative nuclear p65 fold-changes, the median values of the resulting ratios were plotted as Box–Whisker Plots, normalized to the median of 0 h/0 Gy/ + TNF-α−treated replicates [**(A)**, right panel]. **(B)** Box–Whisker Plots of relative nuclear p65 fold changes after X-ray irradiation with 0.5 Gy (light gray boxes) and 6 Gy (dark gray boxes) were normalized to the respective reference values (0 Gy/ + TNF-α, white). All experiments were performed in duplicates.

To address TNF-α-mediated NF-κB activation, we monitored the nuclear level of p65 under static conditions after irradiation treatment. In order to control for possible cell cycle dependent or senescence related variations in the cellular response, the analysis was performed by discriminating different classes of HMVEC (details in Figure S3 in Supplementary Material). As shown in Figure 6A, we defined three distinct subpopulations based on their respective nuclear area and shape: A (42.8% of the nuclei), B (27% of the nuclei), and C (30% of the nuclei). For all subpopulations, 1 h posttreatment TNF-α-stimulated cells revealed a 1.4-fold increase in relative nuclear p65 translocation, indicating NF-κB activation. The nuclear levels of p65 decreased between 3 and 24 h to ~1.2-fold compared to untreated cells (Figure 6A). Although considerable variability between replicates was noticed, all subpopulations demonstrated similar trends. However, as depicted in Figure 6B, combined treatment of HMVEC with TNF-α and irradiation did not result in obvious changes.

## Discussion

In this work, we present an optimized flow chamber device with removable scaffolds for cell growth. These scaffolds are glass cover slips; glass is a well-tolerated substrate for cells and appropriate for further immunofluorescence staining and microscopic analysis. The latter may provide further options of labeling, e.g., by phalloidin, for more detailed analysis of cells, and assessment of senescence or cell death. An advantage of the novel system is the larger growth area compared to most commercially available systems [parallel plate flow systems (42, 43)]. The flexibility of the scaffold renders the device particularly suitable for experiments with special geometrical requirements, i.e., for irradiation exposure of cells at charged particle accelerators. This allows for the cultivation of cells under laminar conditions prior and after irradiation on the same scaffold. In addition, we have framed a work flow, including a semiautomated quantification of adherent leukocytes, allowing for a higher throughput compared to manual counting and less biased analysis of the data. As a more general perspective, this system allows for different types of *in vitro* investigations of vascular effects closer to physiological conditions than under static conditions.

Using laminar culture conditions for human primary EC, we detected a lower basal level of PBL adhesion compared to static conditions (Figures 2A,B), which was maintained in the presence of TNF-α. This result is in agreement with published data, showing that shear stress reduces adhesion (10) and TNF-α-mediated inflammatory reactions of EC, i.e., the adhesion related expression of adhesion molecules (E-Selectin, ICAM-1, and VCAM-1) and chemokines [IL-8, MCP-1 (40, 44)]. Notably, the elongated morphology of the EC cultivated under laminar conditions (Figure 2C) further corresponds to observations reported by others (44, 45) and is probably related to a changed expression of genes involved in cell–matrix interactions (46).

The major purpose of our study, when constructing a flow chamber system was to unravel potential anti-inflammatory effects of low doses of densely ionizing charged particles compared to X-rays. It is known for decades that patients with chronic inflammatory diseases can benefit from low-dose photon irradiation (15, 17, 36), but the underlying mechanisms are not fully resolved, especially for α-particle irradiation via Radon exposure (12). To address this issue, we used He-ion irradiation which has nearly identical physical characteristics and was used instead of α-particles resulting from radioactive decay. The read-out for an anti-inflammatory response was the level of PBL adhesion to irradiated EC.

In previously published work, the adhesion of mononuclear cells, leukocytes, or immortalized cell lines to EC has been investigated under static conditions or non-linear shear stress. A lowered adhesion was found after low dose X-ray exposure of EC compared to non-irradiated cells (11, 27, 36). For these studies, predominantly established lines like the hybrid cell line EA.hy926 were used, which are considered to display characteristics of primary EC. In a first step, using static conditions, we could confirm the reported lowered adhesion of PBL to EA.hy926 after exposure to low X-ray doses, evaluated by flow cytometric quantification of stained PBL (Figure S1 in Supplementary Material). However, adhesion to EA.hy926 cells might be influenced by the tumor component (A549) of this hybrid cell line. Therefore, we used primary human EC isolated from dermal microvasculature (HMVEC) for the following investigations under laminar conditions. Laminar conditions represent an additional modification to the original protocols where “non-linear shear conditions” were applied (11).

We observed for primary cells a trend for decreased adhesion to TNF-α-stimulated HMVEC after X-ray exposure, especially when applying laminar culture conditions (Figures 3C,D). Under static conditions, the radiation effects were less pronounced than for EA.hy926 cells (Figure S1 in Supplementary Material) and those reported previously (11). The major interexperimental variations may arise from the use of PBL, which for technical reasons were isolated from blood of different donors with an individual immune status in each experiment. Of note, the radiation-induced decrease in adhesion was only significant under laminar conditions. We hypothesize that this is caused by the “stringent conditions” under laminar flow, leaving PBL only attached to the endothelial layer if tight binding between both cell types occurred.

The results obtained for He-ion exposure endorse this interpretation (Figure 4). It seems likely that under static conditions densely ionizing irradiation does not result in a decrease in adhesion, while this was demonstrated under laminar conditions. In contrast to X-ray exposure, this accounted only for the lowest dose (0.1 Gy), where, according to the Poisson distribution, the probability for a charged particle to traverse a cell nucleus is 80% (details in Materials and Methods section). This preliminary result points to a comparable anti-inflammatory effect after exposure to densely ionizing He-ions as shown for X-rays. This is a first step to elucidate the mechanisms underlying the effects evoked by the α-particle emitter radon used for the treatment of chronic inflammatory diseases (12, 47–49).

The question which molecular changes are involved in the adhesion to TNF-α-stimulated primary EC cannot be answered yet. Our recently published results for non-laminar shear stress indicate an involvement of intracellular reactive oxygen species (ROS) inhibiting the adhesion of leukocytes. This is related to the cellular ROS defense, which is not fully activated at lower doses (50). Interestingly, ROS and NO signaling as well as the ROS detoxifying system are reported to be changed under laminar conditions (40, 51).

We also investigated the expression of adhesion molecules under static conditions. As expected, the levels of adhesion molecules in HMVEC were clearly enhanced upon TNF-α stimulation (52, 53), but not modified by additional irradiation (Figure 5), suggesting activation rather than increased expression of adhesion molecules as the major mediator of radiation induced changes of adhesion. Of note, on the surface of HMVEC, ICAM-1, and VCAM-1 were expressed and E-Selectin was not detectable. In contrast, EA.hy926 cells expressed ICAM-1 and E-Selectin, were both enhanced upon TNF-α stimulation, but not modified by irradiation and VCAM-1 was not detectable (Figure S2 in Supplementary Material), indicating differences in the molecular basis of the adhesion process between both primary and hybrid EC. In line with the proposed activation of NF-κB pathway in the adhesion process, the nuclear translocation of the p65 subunit was found upon TNF-α stimulation of HMVEC, but not after irradiation under static conditions (Figure 6). Here, a detailed analysis of subpopulations with different nuclear morphology was performed, taking into account the apparent heterogeneity of the EC population [Figure 2B (54)], but no obvious differences for the subpopulations were found.

In summary, we here report on a novel flow chamber system, which can be used for assessment of leukocyte adhesion to EC in general. While the radiation response of TNF-α-stimulated primary EC under static conditions is variable, we could show more robust radiation induced changes in adhesion under laminar conditions, also for densely ionizing helium ion exposure. In further studies, the molecular basis, i.e., expression and/or activation/clustering of adhesion molecules and NF-κB signaling, will be addressed under laminar conditions.

## Author Contributions

NE and FRa contributed equally to this work. NE, FRa, PW, CF, BB, CC, and FRö contributed to the conception. NE, FRa, CF, SH, FRö, BB, and CC designed the work. NE, FRa, SK, ASB, TD, BB, SM, SH, and TF performed the acquisition and analysis. NE, FRa, SK, CF, SH, FRö, MD, TF, BB, and CC contributed to the interpretation of data for the work. All authors contributed to drafting the work or revising it critically for important intellectual content, final approval of the version to be published, and agreement to be accountable for all aspects of the work in ensuring that questions related to the accuracy or integrity of any part of the work are appropriately investigated and resolved.

## Conflict of Interest Statement

The authors declare that the research was conducted in the absence of any commercial or financial relationships that could be construed as a potential conflict of interest.
