# Supplementary material for: Measuring Leukocyte Adhesion to (Primary) Endothelial Cells after Photon and Charged Particle Exposure with a Dedicated Laminar Flow Chamber
Source: Front Immunol. 2017 Jun 1;8:627. doi: 10.3389/fimmu.2017.00627 (PMC5451490; doi:10.3389/fimmu.2017.00627)
Supplement: Supplementary file 1 [file presentation_1.pdf]

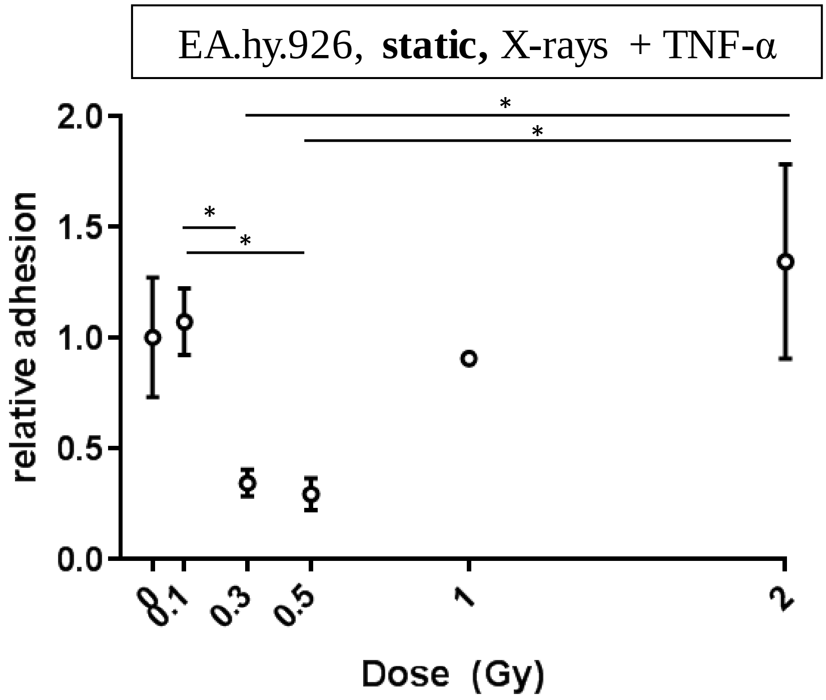

Figure S1: Adhesion of PBL to hybrid endothelial cells (EA.hy.926) after combined treatment with TNF-α and X-irradiation. EA.hy.926 were cultivated under *static* conditions, stimulated with 1 ng/ml TNF- α and irradiated with X-rays (0.1 to 2 Gy). After 24 h, PBL were co-cultivated with EA.hy.926 under *static* conditions and adherent PBL were evaluated by flow cytometric quantification of PKH26-stained PBL. N= 2, at least triplicates were analyzed. Mean +/- SEM. T-test was applied $p < 0.05$ was considered as significantly different from the values indicated by connecting lines and (*).

A decreased PBL adhesion is observed at low doses of 0.3 and 0.5 Gy, which is in accordance with previously published data (Roedel et al., 2002; Hildebrandt et al., 2002). At doses above 1 Gy, PBL adhesion is not changed compared to reference values (0 Gy +TNF-α), or higher (2 Gy).

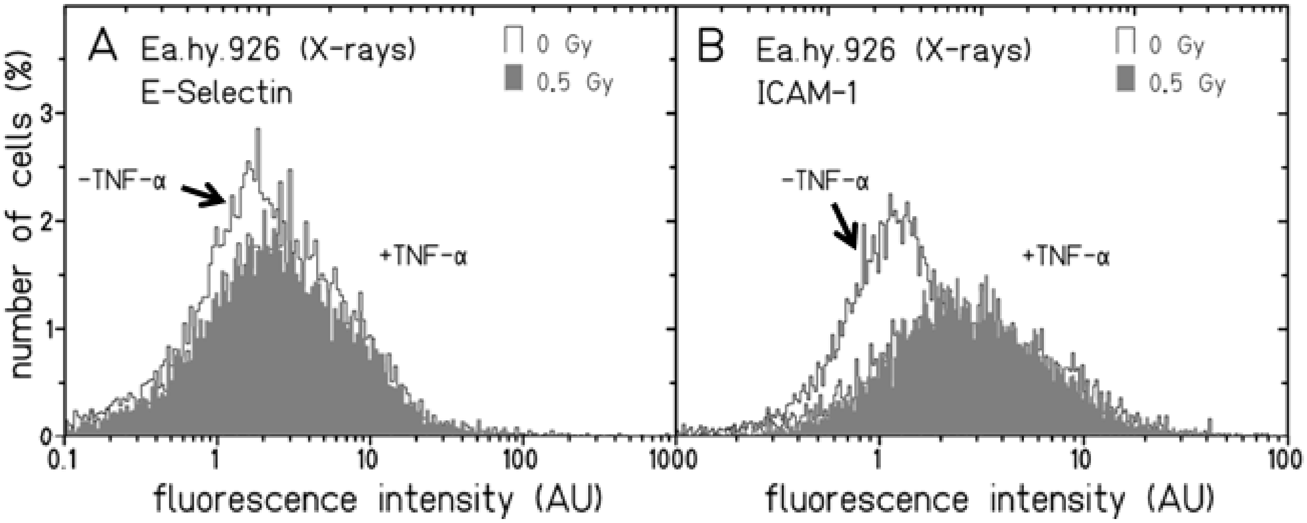

Figure S2: Expression of adhesion molecules on the cellular surface of endothelial hybrid (Ea.hy926) cells.

Representative distributions of fluorescence intensities for adhesion molecules in EA.hy.926 cells, cultivated under *static* conditions for 48 h, and then treated either without (-TNF-α) or with (1ng/ml TNF-α; white curves) and X-irradiated (0.5 Gy, grey curves). E-Selectin (A) and ICAM -1 (B) expression is shown (N=4, triplicates).

TNF-α stimulation results in a small increase, but additional X-irradiation with 0.5 Gy does not further change the expression level. The curves for 0 Gy/+TNF-α and 0.5 Gy/+TNF-α are almost completely overlapping.

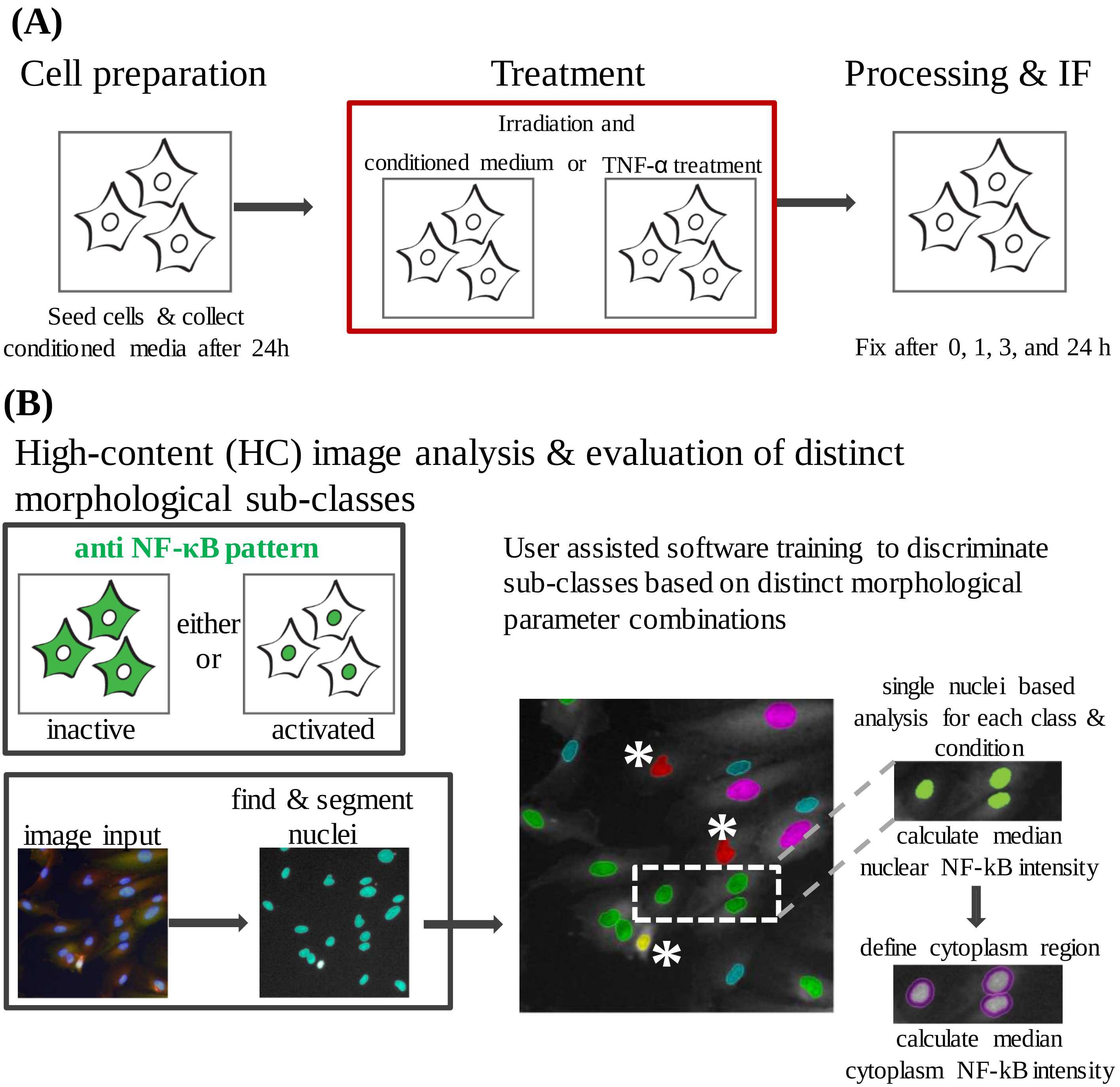
(A)
Cell preparation
Treatment
Processing & IF
Irradiation and
conditioned medium or TNF-α treatment
Seed cells & collect
conditioned media after 24h
Fix after 0, 1, 3, and 24 h
(B)
High-content (HC) image analysis & evaluation of distinct
morphological sub-classes
anti NF-κB pattern
either
or
inactive
activated
User assisted software training to discriminate
sub-classes based on distinct morphological
parameter combinations
image input
find & segment
nuclei
single nuclei based
analysis for each class &
condition
calculate median
nuclear NF-kB intensity
define cytoplasm region
calculate median
cytoplasm NF-kB intensity

Figure S3: High-content (HC) image analysis procedure to monitor the activation of NF-κB after TNF-α treatment and irradiation.

Before irradiation and TNF-α treatment, cells were seeded and allowed to condition the cell culture medium. Prior to irradiation, the conditioned medium was collected and samples were irradiated either with 0, 0.5 or 6 Gy X-rays. Immediately after irradiation, samples were either cultured in conditioned medium or conditioned medium supplemented with 1 ng/ ml TNF-α, for 0, 1, 3 or 24 h, respectively.

At given time-points, samples were fixed and processed for immunofluorescent detection of the NF-κB subunit p65. In addition nuclear and cytoplasm compartments were highlighted using DAPI-DNA counterstaining and F-actin labeling via phalloidin (A). High-content (HC) imaging was used to analyze relative nuclear NF-κB fold-changes.

To this end, immunofluorescent (IF) detection of p65 in combination with actin (cytoplasm marker) and DAPI-staining (nuclear marker) was used to monitor the activation state of p65 (inactive: absence of nuclear signals; active: nuclear p65 signals). Resulting samples were analyzed in an HC imaging approach combined with a user-assisted classification algorithm that allowed to discriminate sub-populations based on nuclear morphology parameters (B). Starting with an input image, nuclei were identified by using DNA-DAPI signals ("find nuclei", in-built method "M"). Morphological properties were calculated (e.g. roundness, nuclear size, width, length, ratio of width and length, etc.) and collected parameters were utilized to discriminate distinct nuclear morphologies. Therefore, the software algorithm was user-assisted trained with the definition of at least 30 cells per class. After exclusion of large (red) and small (yellow) artefacts (asterisks, e.g unsegmented/ over segmented nuclei), three morphological classes (class A: medium size nuclei, rather oval than round; class B: large to very large nuclei, rather round or oval; class C: smaller than class A nuclei, rather elongated or oval) were further analyzed. (B, left panel).

After classification, intensity properties of p65 signals were calculated for each nucleus and condition. To obtain relative nuclear fold-changes of p65 intensity, an individual ring region around the nucleus with an outer border of -7 and an inner border of 1 pixel was defined as cytoplasm area (A_{cyto}). Median intensity values of nuclear area (I (A_{nuc})) and cytoplasm region (I (A_{cyto})) were used to calculate relative nuclear p65 fold-changes for each cell, class and condition. The resulting numeric values obtained from the "Harmony" analysis software were fed into the open source software "R" (http://www.R-project.org/) for further statistical analysis and visualization. Finally, Box-Whisker-Plots were generated for each morphological class and treatment, showing the relative nuclear fold-change normalized to unirradiated controls.
